# Safety of anterior cervical corpectomy and fusion (ACCF) for the treatment of subaxial cervical spine injuries, a single center comparative matched analysis

**DOI:** 10.1007/s00701-024-06172-1

**Published:** 2024-07-03

**Authors:** Victor Gabriel El-Hajj, Aman Singh, Alexander Fletcher-Sandersjöö, Simon Blixt, Vasilios Stenimahitis, Gunnar Nilsson, Paul Gerdhem, Erik Edström, Adrian Elmi-Terander

**Affiliations:** 1 Department of Clinical Neuroscience, Karolinska Institutet, Stockholm, Sweden; 2 Capio Spine Center Stockholm, Löwenströmska Hospital, 194 02 Upplands-Väsby, Box 2074, Stockholm, Sweden; 3 Clinical Science, Intervention and Technology (CLINTEC), Karolinska Institutet, Stockholm, Sweden; 4 Department of Surgical Sciences, Uppsala University, Uppsala, Sweden; 5 Department of Hand Surgery and Orthopedics, Uppsala University Hospital, Uppsala, Sweden

**Keywords:** Subaxial cervical spine injury, ACDF, ACCF, Corpectomy, Complications, Safety

## Abstract

**Introduction:**

Anterior Cervical Discectomy and Fusion (ACDF) and Anterior Cervical Corpectomy and Fusion (ACCF) are both common surgical procedures in the management of pathologies of the subaxial cervical spine. While recent reviews have demonstrated ACCF to provide better decompression results compared to ACDF, the procedure has been associated with increased surgical risks. Nonetheless, the use of ACCF in a traumatic context has been poorly described. The aim of this study was to assess the safety of ACCF as compared to the more commonly performed ACDF.

**Methods:**

All patients undergoing ACCF or ACDF for subaxial cervical spine injuries spanning over 2 disc-spaces and 3 vertebral-levels, between 2006 and 2018, at the study center, were eligible for inclusion. Patients were matched based on age and preoperative ASIA score.

**Results:**

After matching, 60 patients were included in the matched analysis, where 30 underwent ACDF and ACCF, respectively. Vertebral body injury was significantly more common in the ACCF group (*p* = 0.002), while traumatic disc rupture was more frequent in the ACDF group (*p* = 0.032). There were no statistically significant differences in the rates of surgical complications, including implant failure, wound infection, dysphagia, CSF leakage between the groups (*p* ≥ 0.05). The rates of revision surgeries (*p* > 0.999), mortality (*p* = 0.222), and long-term ASIA scores (*p* = 0.081) were also similar.

**Conclusion:**

Results of both unmatched and matched analyses indicate that ACCF has comparable outcomes and no additional risks compared to ACDF. It is thus a safe approach and should be considered for patients with extensive anterior column injury.

**Supplementary Information:**

The online version contains supplementary material available at 10.1007/s00701-024-06172-1.

## Introduction

Surgery has a fundamental role in the management of subaxial cervical spine injuries. Depending on the various mechanisms and extent of injury, a range of surgical methods and approaches may be used. Anterior Cervical Discectomy and Fusion (ACDF) and Anterior Cervical Corpectomy and Fusion (ACCF) are both viable surgical approaches in the management of pathologies of the cervical spine [3, 11, 17, 19, 26, 28]. Although both largely established in spine surgery [23, 25], there are no clear guidelines surrounding the use of one or the other of the techniques in the setting of subaxial traumatic injuries. ACCF offers improved visualization of the injury site compared to ACDF [9] and recent reviews have demonstrated that ACCF provides better decompression results [7, 8, 28]. Nonetheless, certain reports indicate a higher perioperative complication risk associated with ACCF as compared to ACDF [18, 27]. Additionally, ACCF is more technically challenging, and its use within the context of cervical spine trauma is limited and poorly researched. In contrast, the use of ACDF for subaxial spinal injuries has been widely described within the literature. This discrepancy may arise from a reluctance to utilize ACCF due to its more invasive and risk-bearing profile.

Regardless of the approach, the goals of surgery remain decompression of the spinal cord, reduction and stabilization of the injured segment [2], and maintenance of cervical lordosis [13]. Adequate decompression of the spinal cord is essential for the prevention of increased intramedullary pressure with resulting reductions in the perfusion pressure, especially in the setting of a posttraumatic medullary edema [6, 8, 14].

In this retrospective single center study, data for all patients undergoing ACDF or ACCF for subaxial cervical spine injuries involving the fusion of three vertebrae, was reviewed. The aim of the study was to compare procedural and periprocedural complications and outcomes following ACDF or ACCF for the treatment of subaxial cervical spine injuries. This comparison seeks to delineate risk profile differences between ACCF and the more commonly used ACDF.

## Methods

### Patient selection

This study complies with all ethical guidelines and regulations and was approved by the Swedish Ethical Review Authority. The study hospital is a publicly funded tertiary care center serving a region of approximately 2 million inhabitants. It is the region’s only level 1 trauma center and handles most of the spinal trauma cases in the region. Patients were identified through the surgical management software Orbit (Evry Healthcare Systems, Solna, Sweden). Medical records and imaging data from digital hospital charts were retrospectively reviewed using the health record software TakeCare (Compu Group Medical Sweden AB, Farsta, Sweden). The need for patient informed consent was waived, as per Swedish regulations on the use of retrospective patient data. The inclusion criteria were subaxial traumatic cervical spine injury, treated with 2-level ACDF or 1-level ACCF (surgeries spanning over 2 discs and fusing 3 vertebrae). The exclusion criteria were degenerative cases, non-traumatic cases, traumatic cases primarily treated with posterior or anteroposterior surgery, and cases with incomplete records. A total of 629 adult patients treated with ACDF or ACCF during the period of 2006 to 2018 were screened and 104 cases (54 ACDF and 50 ACCF) were included in the study. Preoperative diagnostic imaging included, in the vast majority, an initial trauma CT scan followed by an MRI. The cohort of patients undergoing ACDF was considered as the control group, given the established nature of the procedure in a traumatic context, as opposed to the ACCF.

### Surgical technique

All surgeries were performed by at least one attending senior neurosurgeon. A standard right-sided Smith-Robinson approach was performed in all cases.

ACDF: following discectomy and osteophyte removal, decompression was performed using a high-speed drill at both levels. The posterior longitudinal ligament was typically not opened unless disrupted due to the trauma. PEEK cages were used in all cases.

ACCF: following discectomy above and below the intended vertebrae, corpectomy was performed using a high-speed drill. The posterior longitudinal ligament was typically removed. When using a titanium mesh cage (TMC), as done in 41 of the cases, a cage of appropriate dimensions was chosen, filled with the salvaged bone from the vertebra, and placed in the corpectomy defect. Expandable PEEK cages were otherwise used in six, and iliac crest autograft in three cases.

Adequate alignment and correct position of the cage was confirmed by fluoroscopy. An anterior plate was then positioned, bridging the vertebrae above and below the cage(s) and stabilized with bicortical screws under fluoroscopic guidance.

### Postoperative follow-up

Patients were mobilized without collars after surgery. Postoperative clinical controls and a CT-scan were usually performed within the first 24 h. In adherence with routine protocols, all patients underwent follow-up CT scans at approximately 4 weeks and 3 months after initial surgery. All patients were clinically evaluated by their surgeon after 3 months. The median time to short-term follow-up in this cohort was 3 months (IQR: 1–5). Additional imaging, in selected cases, was performed when clinically indicated. The last clinical follow-ups were performed at a median of 76 months postoperatively.

### Statistics and matching

For descriptive purposes, categorical data are presented as number (proportion). The normality of continuous data was evaluated using the Shapiro–Wilk test. Since the distribution of all continuous data deviated significantly from a normal distribution pattern, medians with interquartile ranges (IQR) were used. Variables potentially interfering with the primary study outcome were identified based on previous studies. Case–control matching was then used to account for these potential confounders. Variables that were included in the matching process were age and the preoperative ASIA score, with degrees of freedom of 5 and 0, respectively. Owing to the inherent distinctions in surgical indications between ACDF and ACCF, there is a potential overrepresentation of more severe and extensive injuries among patients undergoing ACCF. This could introduce bias into the results, potentially exaggerating the surgical risks associated with this procedure. Therefore, the process of matching based on the aforementioned variables aims to establish cohorts with comparable severity of injuries, mitigating the impact of these underlying differences. Statistical significance was set to *p* < 0.05. Statistical analyses were conducted using SPSS and R.

## Results

### Unmatched analysis

In total 629 patients were screened and 104 patients undergoing ACDF or ACCF, both involving the removal of 2-discs and the fusion of 3 vertebrae, were identified and included in the unmatched analysis (Table 1). There was no significant difference in male sex distribution between ACDF and ACCF groups (70% vs 74%, *p* = 0.680). Patients in the ACDF group were significantly older than those in the ACCF group (62.9 vs 39 years, *p* < 0.001). No significant difference in body mass index (BMI) was found between the groups (25 vs 24, *p* = 0.122). Regarding trauma mechanism, the ACDF group had a higher proportion of patients experiencing fall from height (36% vs. 30%) and road traffic accidents (30% vs. 16%) compared to the ACCF group (*p* = 0.043).

**Table 1:** Baseline and radiographic characteristics of patients in both anterior cervical discectomy (ACDF) or corpectomy (ACCF) and fusion groups

	Unmatched analysis			Matched analysis		
Baseline variables	ACDF (*n* = 54)	ACCF (*n* = 50)	*p*-value	ACDF (*n* = 30)	ACCF (*n* = 30)	*p*-value
Male sex	38 (70%)	37 (74%)	0.680	19 (63%)	21 (70%)	0.584
Age	62.9 (24)	39 (25)	** < 0.001**	49 (29)	42 (27)	0.220
BMI	25 (5)	24 (5)	0.122	24 (5)	24 (6)	0.987
Trauma mechanism			**0.043**			0.115
Fall from height	16 (30%)	18 (36%)		9 (30%)	12 (40%)	
Motor vehicle accident	16 (30%)	8 (16%)		11 (37%)	3 (10%)	
Same-level fall	14 (26%)	7 (14%)		5 (17%)	6 (20%)	
Hit by car	0 (0%)	4 (8%)		0 (0%)	2 (7%)	
Bike accident	0 (0%)	2 (4%)		0 (0%)	0 (0%)	
Other	8 (15%)	11 (22%)		5 (17%)	7 (23%)	
Preoperative ASIA score			0.068			> 0.999
E	21 (39%)	19 (38%)		11 (37%)	11 (37%)	
D	20 (37%)	14 (28%)		11 (37%)	11 (37%)	
C	5 (9%)	3 (6%)		3 (10%)	3 (10%)	
B	6 (11%)	3 (6%)		3 (10%)	3 (10%)	
A	2 (4%)	11 (22%)		2 (7%)	2 (7%)	
Radiology						
Traumatic disc rupture	41 (76%)	25 (50%)	**0.006**	23 (77%)	15 (50%)	**0.032**
Vertebral body injury	28 (52%)	47 (94%)	** < 0.001**	16 (53%)	27 (90%)	**0.002**

*ASIA* American spinal injury association

Radiologically, a significant difference was found in the occurrence of traumatic disc ruptures between ACDF and ACCF groups (76% vs 50%, *p* = 0.006). Vertebral body injuries were, however, more common in the ACCF than the ACDF group (90% vs 52%, *p* < 0.001).

No significant differences were found in surgical complication rates, including wound infection, cerebrospinal fluid (CSF) leak, dysphagia, other complications, and the composite outcome for any complication, between ACDF and ACCF groups (Table 2).

**Table 2:** Surgical complications and outcomes in in both anterior cervical discectomy (ACDF) or corpectomy (ACCF) and fusion groups

	Unmatched analysis			Matched analysis		
Complications and outcomes	ACDF (*n* = 54)	ACCF (*n* = 50)	*p*-value	ACDF (*n* = 30)	ACCF (*n* = 30)	*p*-value
Wound infection	1 (2%)	1 (2%)	> 0.999	1 (3%)	1 (3%)	> 0.999
CSF leak	0 (0%)	1 (2%)	0.481	0 (0%)	1 (3%)	> 0.999
Dysphagia	3 (6%)	3 (6%)	> 0.999	1 (3%)	1 (3%)	> 0.999
Other	0 (0%)	2 (4%)	0.229	0 (0%)	2 (7%)	0.492
Composite outcome for any complication	4 (7%)	6 (12%)	0.515	2 (7%)	4 (13%)	0.671
Long-term ASIA score	3 missing	2 missing	0.319	2 missing	1 missing	0.081
E	25 (46%)	30 (60%)		12 (41%)	20 (71%)	
D	18 (33%)	7 (14%)		12 (41%)	6 (21%)	
C	3 (6%)	3 (6%)		1 (3%)	2 (7%)	
B	3 (6%)	3 (6%)		2 (7%)	0 (0%)	
A	2 (4%)	4 (8%)		2 (7%)	0 (0%)	
Fixation failure requiring supplementary posterior fixation	2 (4%)	2 (4%)	> 0.999	1 (3%)	2 (7%)	> 0.999
90-day mortality	0 (0%)	1 (2%)	0.481	0 (0%)	1 (3%)	> 0.999
1-year mortality	2 (4%)	1 (2%)		2 (7%)	1 (3%)	> 0.999

*ASIA* American spinal injury association

At long-term follow-up (median: 76 months; IQR: 76), there was no significant difference in the distribution of ASIA scores between the ACDF and ACCF groups (*p* = 0.319). There were no deaths recorded within the 30-day postoperative period, and only one death in the 90-day postoperative period. The patient who died had received an ACCF, but the death was unrelated to the procedure. In total, three patients died within one year of their injury, two of whom received an ACDF, and one an ACCF.

## Matched analysis

After matching based on age and preoperative ASIA score, an analysis was performed comparing 30 cases with each approach (Table 1). There was no significant difference in the distribution of male sex (63% vs 70%, *p* = 0.584), age (49 vs 42 years, *p* = 0.220), BMI (24 vs 24, *p* = 0.987), trauma mechanism (*p* = 0.115), or preoperative ASIA scores between the two groups (*p* > 0.999).

Radiologically, significant differences were observed in the occurrence of traumatic disc rupture (ACDF: 77%, ACCF: 50%, *p* = 0.032) and vertebral body injury (ACDF: 53%, ACCF: 90%, *p* = 0.002). Surgery was performed on average within 48 h of the trauma, without any significant difference between the groups (*p* = 0.055).

Surgical complications, including wound infection, CSF leak, dysphagia, other complications, and the composite outcome for any complication, did not show significant differences between the ACDF and ACCF groups. The occurrence of construct failure and supplementary posterior fixation did not differ between the groups (*p* = 0.990).

No significant difference was found in the distribution of long-term (median: 76 months; IQR: 92) ASIA scores between ACDF and ACCF groups (*p* = 0.081), or the postoperative mortality rates (Table 2).

**Fig. 1:**
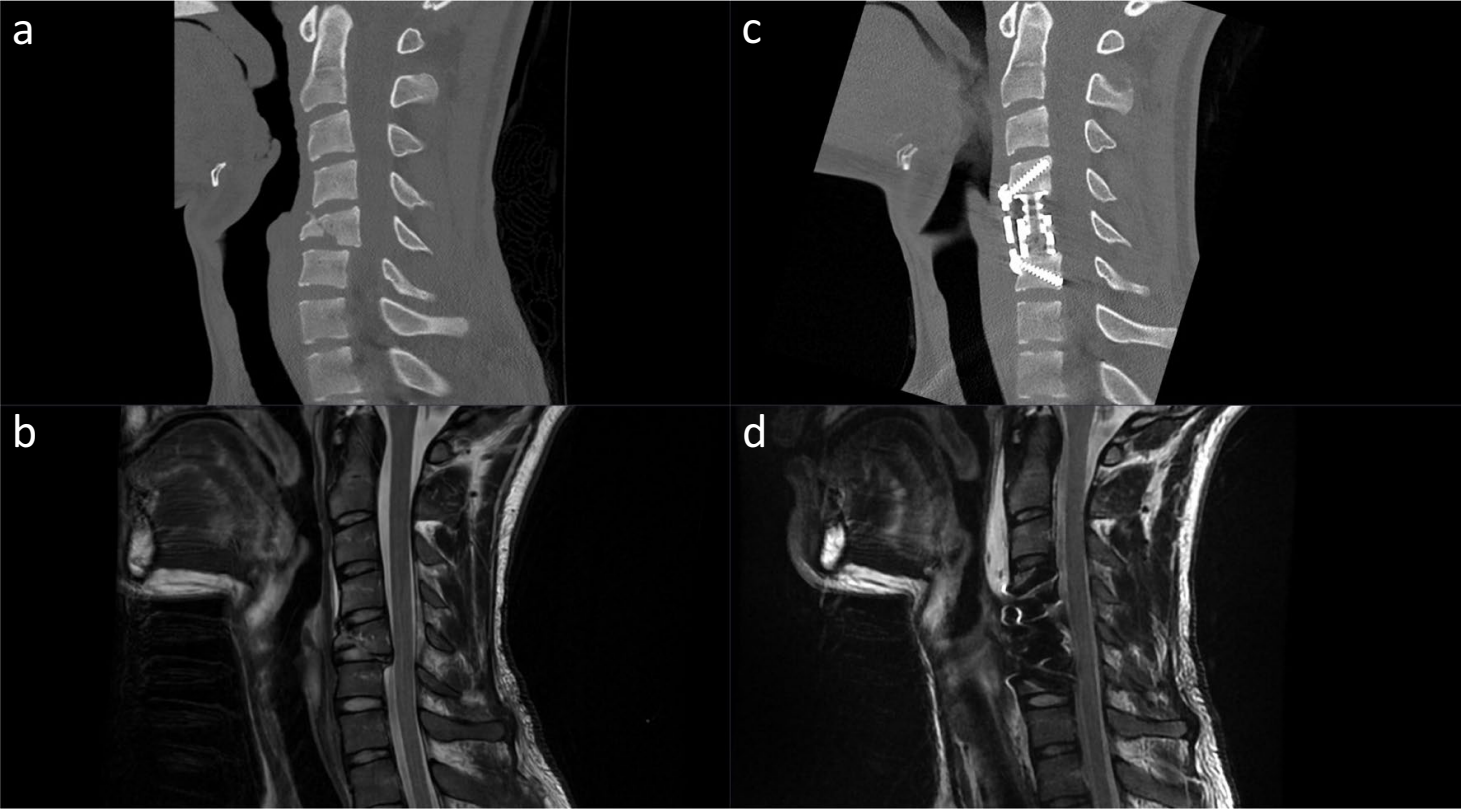
Preoperative CT (**A**) and MRI (**B**) as well as postoperative CT (**C**) and MRI (**D**) of a patient undergoing a 1-level anterior corpectomy and fusion (ACCF)

**Fig. 2:**
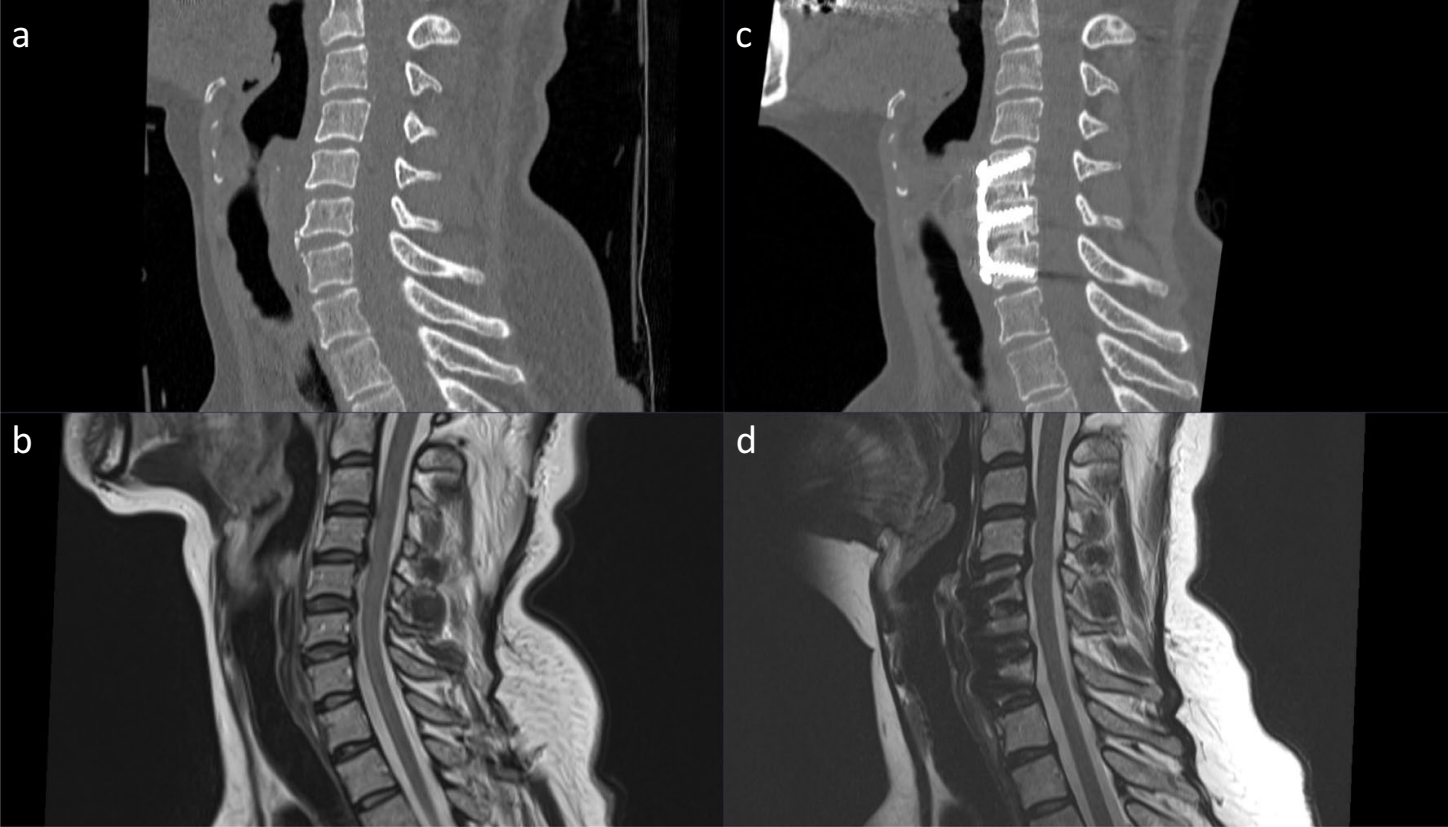
Preoperative CT (**A**) and MRI (**B**) as well as postoperative CT (**C**) and MRI (**D**) of a patient undergoing a 2-level anterior discectomy and fusion (ACDF)

Pre- and postoperative CT and MRI imaging of two patients treated with 1-level ACCF and 2-level ACDF, respectively, is provided (Figs. 1 and 2).

## Discussion

In this study, we reviewed our experience with two-level ACDF vs one-level ACCF for the treatment of subaxial cervical spine injuries, contrasting the two techniques regarding complications and outcomes. As expected, ACCF was more commonly performed in patients with vertebral body injury (*p* = 0.002), and ACDF in those with traumatic disc rupture (*p* = 0.032). With greater severity of injury, i.e. vertebral body injury, ACCF was favored. ACCF is known to be technically more challenging and carries a higher risk of complications, such as injury to the dura, spinal cord, or nerve roots [10, 15, 21, 30]. However, in this study the complications and clinical outcomes did not significantly differ between ACDF and ACCF groups. This is in line with previous literature on degenerative cervical spine surgery, which revealed similar outcomes and complication rates between one-level ACCF and two level ACDF. The only differences seen between the two procedures concerned operative time, blood loss, and length of hospital stay, all in favor of ACDF [5, 12, 22].

Evidence provided by studies on degenerative cervical spine suggest a higher degree of decompression achieved with ACCF compared to ACDF [1, 10, 15, 24, 24]. This should arguably be the case even in the context of traumatic cervical spine injuries, although very few studies describe the role of ACCF in the management of cervical spine injuries. Nonetheless, our findings provide evidence for the safety of ACCF in the treatment of subaxial cervical spine injuries. ACCF provided adequate decompression of the spinal cord and comparable neurological outcomes to ACDF, without any additional complications. In a nationwide matched analysis comparing ACDF to ACCF based on data from the National Surgical Quality Improvement Program (NSQIP), the authors reached similar conclusions and suggested the use of the procedure that best accomplishes the surgical objectives [5]. Interestingly, in that study, ACCF was associated with major postoperative adverse events. This association, however, was later revoked, after adjusting for operative time, where the operative time was a larger risk factor for complications than the approach itself [5]. Efforts should be made to select the right patients, and surgeon, for the procedure, rather than avoiding ACCF for fear of its risk profile.

In fact, a gap in the literature regarding the use of ACCF in the treatment of subaxial cervical spine injuries was detected as only a few studies with limited sample sizes were found. More studies analyzing the outcomes of ACCF in the treatment of cervical spine injuries are warranted to increase the understanding of the risks and benefits of the procedure.

In their series of 99 patients, Madan et al. found ACCF to yield good clinical outcomes regarding postoperative disability and neurological function. Most of the patients (90%) reported mild to no disability on the neck disability index (NDI) and 59% scored ASIA E at last follow-up (vs. 34% on admission). No patients experienced any neurological deterioration [2]. Similarly, in our study most patients (60%) had a long-term postoperative ASIA score of E (vs. 38% on admission) and no patients experienced deterioration.

In a study from a low resource setting on 14 patients undergoing ACCF for subaxial spine injuries, 58% of the patients experienced neurological improvements postoperatively, which is in line with our results (51%). However, a considerably higher rate of complications was reported compared to our results (57% vs. 12%), most likely owing to the differences in settings [4].

The aim of this study was to investigate if ACCF was associated with a higher complication rate compared to ACDF. In the direct comparison between the two treatments no differences were found. To ensure that the lack of difference was not explained by baseline differences between the groups, a matching was performed based on patient age and admission AIS scores. These variables have repeatedly been identified as outcome predictors in the management of traumatic spine injuries [4]. Matching did not affect the outcome of the comparison between ACCF and ACDF.

While we have not fund increased risks associated with ACCF as compared to ACDF, it must be acknowledged that the indications differ between the two procedures. It could be argued that matching based on neurology disregards the more extensive vertebral body injuries associated with spinal cord injury on the one hand and uncomplicated ligamentous injuries treated with ACDF on the other.

Yet, the unmatched analysis failed to reveal noteworthy differences, despite an anticipated higher complication rate within the ACCF-group.

Regardless, the surgeon's decision-making process should prioritize a comprehensive understanding of the underlying pathology rather than solely relying on the statistical risk of complications. This distinction is crucial as the two procedures, while occasionally overlapping, cater to different surgical indications.

Finally, in the context of traumatic cervical spine injuries, there is a need for randomized controlled trials to evaluate the contribution of ACCF relative to ACDF in patients eligible for both approaches.

### Strengths and limitations

This study is, to the best of our knowledge, the first to compare the surgical complications and long-term outcomes following ACCF or ACDF surgeries spanning over 2 discs and fusing 3 vertebrae in the treatment of subaxial cervical spine injuries. Case–control matching was used to account for the effect of potential confounders. The study limitations include its retrospective and single-center design. Moreover, variables such as baseline comorbidity, operative time, blood loss, and length of stay were unavailable and were not accounted for in the comparative analysis. Among all patients screened, only 104 and 60 were included in the unmatched and matched analyses, respectively. This relatively small sample size nonetheless represents the largest cohort to date on ACCF for traumatic injuries. Finally, an important limitation has to do with the generalizability of the findings, given the fact that all procedures were performed in a highly specialized center with extensive experience with both procedures.

## Conclusion

In this study, subaxial cervical spine injuries treated with either ACDF or ACCF spanning over two disc-spaces and three vertebral levels were compared. Despite a greater injury severity in patients receiving ACCF, the frequency of postoperative complications and instrument failure did not differ between approaches. Treatment with ACCF demonstrated comparable long-term neurological outcomes to ACDF. This study provides evidence suggesting similar risk profiles between ACCF and ACDF. In the hands of experienced surgeons, ACCF is a relatively safe approach without increased risk of morbidity and warrants consideration for patients with extensive vertebral injury.

## Supplementary Information

Below is the link to the electronic supplementary material.

Supplementary file1 (DOCX 17 KB)

## Data Availability

No datasets were generated or analysed during the current study.
