# Supplementary material for: Safety of anterior cervical corpectomy and fusion (ACCF) for the treatment of subaxial cervical spine injuries, a single center comparative matched analysis
Source: Acta Neurochir (Wien). 2024 Jul 3;166(1):280. doi: 10.1007/s00701-024-06172-1 (PMC11222236; doi:10.1007/s00701-024-06172-1)
Supplement: Supplementary file 1 — Supplementary file1 (DOCX 17 KB) [file 701_2024_6172_MOESM1_ESM.docx]

Supplementary table 1. Preoperative radiographic characteristics associated with the subaxial injury in both pre- and post-matching cohorts.

| Unmatched analysis | | | | Matched analysis | | | |  |
| --- | --- | --- | --- | --- | --- | --- | --- | --- |
| Variables | Overall cohort  (n = 104) | ACCF  (n = 50) | ACDF  (n = 54) | p-value | Overall cohort  (n = 60) | ACCF  (n = 30) | ACDF  (n = 30) | p-value |
| Level of injury |  |  |  | **0.006** |  |  |  | 0.82 |
| C4 | 11 (11%) | 9 (17%) | 2 (4.0%) |  | 4 (7%) | 2 (7%) | 2 (7%) |  |
| C5 | 32 (31%) | 17 (31%) | 15 (30%) |  | 19 (32%) | 10 (33%) | 9 (30%) |  |
| C6 | 44 (42%) | 25 (46%) | 19 (38%) |  | 20 (33%) | 11 (37%) | 9 (30%) |  |
| C7 | 17 (16%) | 3 (5.6%) | 14 (28%) |  | 17 (28%) | 7 (23%) | 10 (33%) |  |
| Myelomalacia | 49 (47%) | 27 (54%) | 22 (41%) | 0.176 | 28 (47%) | 17 (57%) | 11 (37%) | 0.121 |
| ALL injury | 73 (70%) | 31 (62%) | 42 (78%) | 0.790 | 42 (70%) | 19 (63%) | 23 (77%) | 0.260 |
| PLL injury | 35 (34%) | 22 (44%) | 13 (24%) | 0.320 | 21 (35%) | 12 (40%) | 9 (30%) | 0.417 |
| Flavum injury | 39 (38%) | 27 (54%) | 12 (22%) | **< 0.001** | 23 (38%) | 14 (47%) | 9 (30%) | 0.184 |
| Traumatic disc herniation | 66 (64%) | 25 (50%) | 41 (76%) | **0.006** | 38 (63%) | 15 (50%) | 23 (77%) | **0.032** |
| Vertebral body injury | 75 (72%) | 47 (94%) | 28 (52%) | **< 0.001** | 43 (72%) | 27 (90%) | 16 (53%) | **0.002** |
| Traumatic cervical spondylolisthesis | 41 (39%) | 29 (58%) | 12 (22%) | **< 0.001** | 28 (47%) | 19 (63%) | 9 (30%) | **0.010** |
| Facet joint dislocation | 35 (34%) | 24 (48%) | 11 (20%) | **0.003** | 21 (35%) | 12 (40%) | 9 (30%) | 0.417 |
| Facet fracture | 46 (44%) | 28 (56%) | 18 (33%) | **0.030** | 29 (48%) | 18 (60%) | 11 (37%) | 0.071 |
| Laminar fracture | 46 (44%) | 30 (60%) | 16 (30%) | **0.002** | 26 (43%) | 16 (53%) | 10 (33%) | 0.118 |
| Concomitant skull base fracture | 3 (3%) | 2 (4%) | 1 (2%) | 0.607 | 2 (3%) | 1 (3%) | 1 (3%) | > 0.999 |
| Concomitant vertebral artery injury | 7 (7%) | 2 (4%) | 5 (9%) | 0.439 | 6 (10%) | 2 (7%) | 4 (13%) | 0.671 |
